# Optogenetic recruitment of hypothalamic corticotrophin-releasing-hormone (CRH) neurons reduces motivational drive

**DOI:** 10.1038/s41398-023-02710-0

**Published:** 2024-01-08

**Authors:** Caitlin S. Mitchell, Erin J. Campbell, Simon D. Fisher, Laura M. Stanton, Nicholas J. Burton, Amy J. Pearl, Gavan P. McNally, Jaideep S. Bains, Tamás Füzesi, Brett A. Graham, Elizabeth E. Manning, Christopher V. Dayas

**Affiliations:** 1 School of Biomedical Sciences and Pharmacy, College of Health, Medicine and Wellbeing, University of Newcastle, Callaghan, NSW 2308 Australia; 2 Brain Neuromodulation Research Program, Hunter Medical Research Institute, New Lambton Heights, Sydney, NSW 2305 Australia; 3 School of Psychology, University of New South Wales, UNSW, Sydney, NSW 2052 Australia; 4 Hotchkiss Brain Institute, Cumming School of Medicine, University of Calgary, Calgary, AB Canada; 5 Department of Physiology and Pharmacology, Cumming School of Medicine, University of Calgary, Calgary, AB Canada

**Keywords:** Neuroscience, Physiology

## Abstract

Impaired motivational drive is a key feature of depression. Chronic stress is a known antecedent to the development of depression in humans and depressive-like states in animals. Whilst there is a clear relationship between stress and motivational drive, the mechanisms underpinning this association remain unclear. One hypothesis is that the endocrine system, via corticotropin-releasing hormone (CRH) in the paraventricular nucleus of the hypothalamus (PVN; PVN^CRH^), initiates a hormonal cascade resulting in glucocorticoid release, and that excessive glucocorticoids change brain circuit function to produce depression-related symptoms. Another mostly unexplored hypothesis is that the direct activity of PVN^CRH^ neurons and their input to other stress- and reward-related brain regions drives these behaviors. To further understand the direct involvement of PVN^CRH^ neurons in motivation, we used optogenetic stimulation to activate these neurons 1 h/day for 5 consecutive days and showed increased acute stress-related behaviors and long-lasting deficits in the motivational drive for sucrose. This was associated with increased Fos-protein expression in the lateral hypothalamus (LH). Direct stimulation of the PVN^CRH^ inputs in the LH produced a similar pattern of effects on sucrose motivation. Together, these data suggest that PVN^CRH^ neuronal activity may be directly responsible for changes in motivational drive and that these behavioral changes may, in part, be driven by PVN^CRH^ synaptic projections to the LH.

## Introduction

Mood disorders, including depression, represent a major health and economic burden [1]. Depression has a complex etiology and encompasses a variety of symptoms such as anhedonia, despair, and reduced motivation. Unfortunately, these symptoms often impede the pursuit of and participation in treatment for depression [2]. Chronic stress is a key contributor to the onset of depression in humans and can provoke depressive-like behavior in rodents [3–5]. Indeed, hyperactivation of the neuroendocrine arm of the stress response, the hypothalamic-pituitary-adrenal (HPA) axis, has been reported in approximately 50% of individuals with depression [6]. Further, positive clinical outcomes after treatment with antidepressants have been associated with the normalization of stress hormone levels [7, 8]. Similar results have been reported in rodents, with repeated exposure to low doses of corticosterone in rats resulting in a depressive-like behavioral phenotype which was reversed by chronic antidepressant treatment [9, 10].

While there is a strong relationship between impaired motivational drive and stress-related neuroendocrine activation, much of the evidence linking neuroendocrine activation with distinct behavioral changes is correlational [7–9, 11, 12]. Corticotropin-releasing hormone (CRH) neurons in the paraventricular nucleus of the hypothalamus (PVN) constitute the apex of the HPA axis, initiating a hormonal cascade [13] that culminates in glucocorticoid secretion. In rodents, deficits in preference for sucrose as well as other indices, such as reduced sociability, can be replicated by artificially raising glucocorticoids over several weeks to stress-like levels [9, 14], thus implicating glucocorticoids in the manifestation of these depressive-like behaviors. Whilst glucocorticoids have been functionally implicated in depressive-like behaviors, direct evidence of a necessary rather than an associative role for activation of PVN^CRH^ neurons in these behaviors, particularly motivation, is lacking. An alternative hypothesis that has recently gained support is that such stress-induced behaviors do not require endocrine signaling, and that PVN^CRH^ neurons also *directly drive* altered motivational states through synaptic rather than endocrine actions [15]. Interestingly, emotional and physiological stressors robustly activate PVN^CRH^ neurons [16, 17], whereas real-time recording of these neurons using fiber photometry has shown that these cells are inhibited by positive rewards such as sucrose [18]. These data suggest that stress and motivation interact to determine the activity of these neurons. Further, a recent study that directly manipulated PVN^CRH^ neurons using optogenetics showed that acute stress-related behaviors require an excitatory, glutamatergic projection to the lateral hypothalamus (LH) that occurs on a timescale consistent with synaptic rather than endocrine events [19]. In parallel work, we have demonstrated that the LH is an important substrate of chronic stress-induced motivational disturbances [20]. Together these studies suggest a more nuanced role for PVN^CRH^ neurons in motivated behavior, potentially via their projections to brain regions critical for motivation such as the LH or ventral tegmental area (VTA) [21].

Accordingly, here we aimed to test whether directly activating PVN^CRH^ neurons can induce long-lasting motivational disturbances, as assessed by sucrose self-administration, under low and high effort reinforcement conditions in CRH-Cre transgenic mice. We found that repeated optogenetic activation of PVN^CRH^ neurons increased acute stress-like behaviors but produced a long-lasting reduction in motivation for sucrose. This pattern of effects was also observed following direct stimulation of the PVN^CRH^ terminals located in the LH. Together our data provide the first direct demonstration that repeated experimental activation of PVN^CRH^ neurons is sufficient to produce impairments in motivation and suggest that these effects are likely underpinned by PVN^CRH^ neuron projections to brain centers involved in motivation as well as HPA endocrine control.

## Methods

### Ethics statement

All procedures were performed in accordance with the Prevention of Cruelty to Animals Act (2004), under the guidelines of the National Health and Medical Research Council (NHMRC) Australian Code of Practice for the Care and Use of Animals for Experimental Purposes (2013). Animal ethics were approved by The University of Newcastle Animal Ethics Committee.

### Animals

B6(Cg)-CRH^tm1(cre)Zjh^/J (CRH-IRES-Cre) and B6.Cg-Gt(ROSA)26Sor^tm14(CAG-TdTomato)Hze^/J (Ai14; tdTomato) were obtained from Jackson Laboratory (stock numbers 012704 and 007914 respectively). Pairs of homozygous CRH-IRES-Cre and tdTomato mice were crossed to produce heterozygous CRH-IRES-Cre;tdTomato (CRH-Cre::tdTomato) mice. The selective expression of tdTomato in PVN^CRH^ neurons has been previously characterized by our laboratory, consistent with other work [22–24]. Our preliminary work showed that the electrophysiological profile and expression of CRH neurons in the PVN and amygdala to be identical to previous studies [25]. The sample size was based on previous studies using optogenetic stimulation to control PVN^CRH^ neurons [19]. A total of 40 CRH-Cre and CRH-Cre::tdtomato mice were used in all experiments (8-13 weeks old at the beginning of experimentation; 24 females and 16 males; 19 females and 9 males were used for Experiment 1; 5 females and 7 males were used for Experiment 2). An unequal number of male and female mice were used due to stock availability within the colony. No sex differences were observed. In Experiment 1, 5 animals were excluded from the final analysis due to incorrect fiber optic probe targeting. In Experiment 2, stringent exclusion criteria were set for PVN virus and LH probe placements. Accordingly, the number of animals excluded due to incorrect targeting was 15 due to the complexity of bilateral targeting of the LH and PVN viral hit rate. Food and water were available *ad libitum* and all mice were maintained on a reverse 12-h light/dark cycle (0700 lights off).

### Experiment 1: Effect of optogenetic recruitment of PVNCRH neurons on motivation for sucrose, Fos-protein activity and stress-related behaviors

**Fig. 1:**
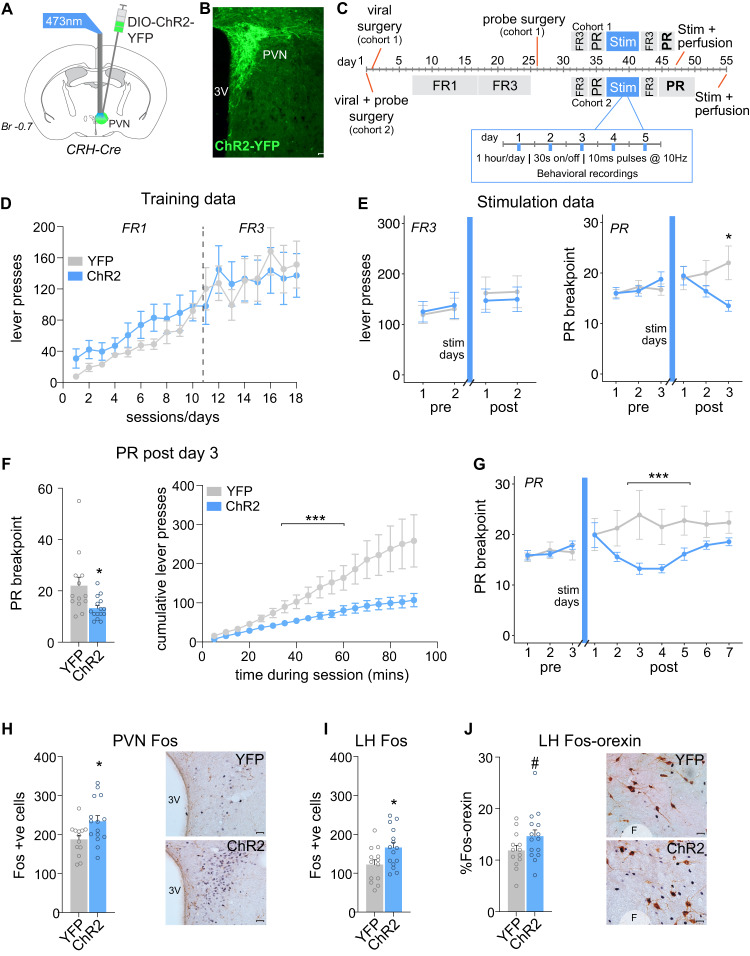
Repeated optogenetic activation of PVN^CRH^ neurons reduces the motivation for sucrose. **A** CRH-Cre transgenic mice were injected with DIO-ChR2-YFP or the YFP control virus, and implanted with a fiber optic probe, above the PVN. **B** ChR2-YFP expression was observed in the PVN. Scale bar, 50 μm. **C** Experimental design for Experiment 1 including timing of operant training, surgery and optogenetic stimulation. **D** There was no difference between mice injected with YFP control virus versus ChR2 virus in the number of active lever presses across FR1 and FR3 training days. **E** There was no effect of repeated PVN^CRH^ photostimulation on the number of active lever presses under a FR3 schedule of reinforcement. Following repeated PVN^CRH^ photostimulation, there was a reduction in PR breakpoint in ChR2 mice compared to controls. *p < 0.05. **F** ChR2 mice had a significant reduction in PR breakpoint on Day 3 post photostimulation, compared to YFP controls. *p < 0.05. This effect was also apparent when lever press data was broken down into 5 min time bins. ***p < 0.001 Time x Virus interaction. **G** The reduction in PR breakpoint following PVN^CRH^ photostimulation in ChR2 mice persisted for up to 7 days post-stimulation, although the effect weakened over time. ***p < 0.001 Day x Virus interaction effect. **H** There was a significant increase in the number of Fos-positive cells in the PVN in ChR2 mice compared to YFP controls following PVN^CRH^ stimulation. *p < 0.05. Scale bar, 25 μm. **I** There was a significant increase in the number of Fos-positive cells in the LH in ChR2 mice compared to YFP controls following PVN^CRH^ stimulation. *p < 0.05. **J** There was a trend towards a significant increase in the percentage of Fos-positive neurons that were orexin positive in the LH in ChR2 mice compared to controls following PVN^CRH^ photostimulation. ^#^*p* = 0.098. Scale bar, 25 μm. PVN paraventricular nucleus of the hypothalamus, LH lateral hypothalamus, PR progressive ratio, FR fixed ratio, ChR2 channelrhodopsin-2, YFP yellow fluorescent protein, stim optogenetic stimulation of PVN^CRH^ neurons, Br bregma, F Fornix.

Refer to Fig. 1A–C for the experimental procedures for Experiment 1. Precise details can be found under the subheadings below. Briefly, mice were randomly allocated to received either YFP control (*n* = 13) or ChR2 (*n* = 15) viral injection surgery targeting the PVN. Fiber optic probe placement occurred during the same surgery in 8 YFP control mice and 9 ChR2 mice (Cohort 2). This was followed by operant training for sucrose under a fixed ratio 1 (FR1) schedule of reinforcement for 10 days then FR3 training for 8 days. Fiber optic probe surgery followed operant training in 5 YFP and 6 ChR2 mice (Cohort 1). All mice were allowed at least 7 days to recover from surgery. Following this, mice were exposed to 2 days of FR3 training and 3 days of progressive ratio (PR) training (termed pre-stimulation training). This was followed by 5 days of chronic PVN^CRH^ optogenetic stimulation consisting of 1 h/day, 10 Hz, 10 ms pulse width, 15 mW, 30 s on 30 s off. During optogenetic stimulation, stress-related behaviors were recorded in each trial for the first 10 min. Following stimulation, mice were split into 2 cohorts, both exposed to 2 FR3 sessions followed by either 3 or 7 PR sessions. Following PR sessions, mice were exposed to a final PVN^CRH^ optogenetic stimulation session and perfused 2 h later for Fos-protein immunohistochemistry.

### Experiment 2: Effect of optogenetic recruitment of the PVN^CRH^ to LH pathway on motivation for sucrose and stress-related behaviors

**Fig. 2:**
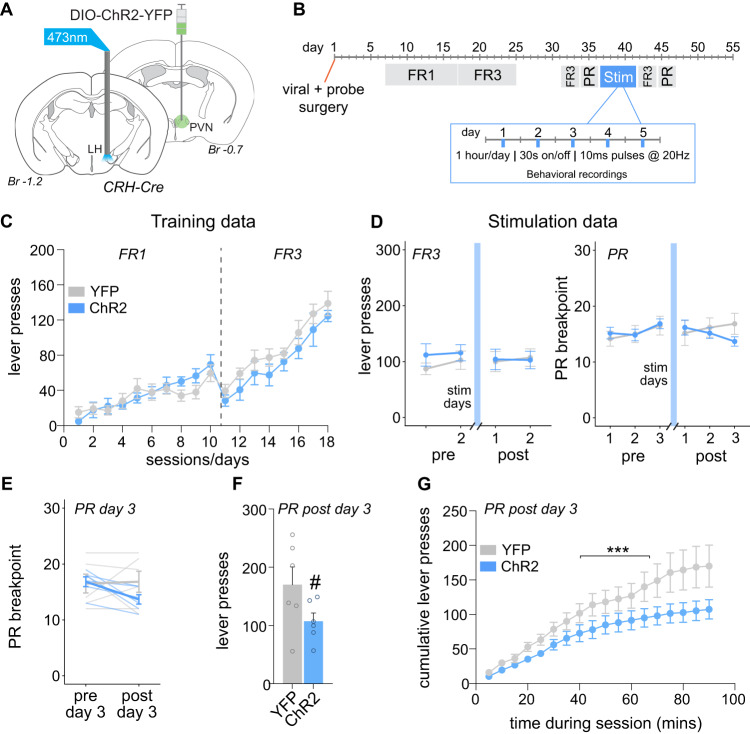
Repeated photostimulation of the PVN^CRH^ to LH pathway modestly reduces the motivation for sucrose. **A** CRH-Cre transgenic mice were injected with DIO-ChR2-YFP or the YFP control virus into the PVN and implanted with fiber optic probes bilaterally in the LH. **B** Experimental design for Experiment 2 including timing of operant training, surgery and optogenetic stimulation. **C** There was no difference between mice injected with YFP control virus versus ChR2 virus in the number of active lever presses across FR1 and FR3 training days. **D** There was no effect of repeated PVN^CRH^ terminal optogenetic stimulation in the LH on the number of active lever presses under a FR3 schedule of reinforcement. There was also no effect of repeated PVN^CRH^ to LH pathway activation on breakpoint under a PR schedule of reinforcement. **E** There was no effect of photostimulation day on PR breakpoint between ChR2 mice and YFP controls. **F** There was a trend towards a significant reduction in the number of PR lever presses in ChR2 mice compared to YFP controls on day 3 post-photostimulation. #p = 0.09. **G** When examining cumulative lever press data on day 3 post-photostimulation for PR, there was a significant reduction in the number of lever presses over time in ChR2 mice compared to YFP controls. ***p < 0.001. PVN paraventricular nucleus of the hypothalamus, LH lateral hypothalamus, PR progressive ratio, FR fixed ratio, stim optogenetic stimulation of PVN^CRH^ to LH pathway, Br bregma.

Refer to Fig. 2A, B for the behavioral procedure for Experiment 2. Precise details of procedures are described below. Briefly, mice were randomly allocated to received either YFP control (*n* = 6) or ChR2 viral injection surgery (*n* = 6) targeting the PVN. In the same surgery, mice were implanted with fiber optic probes targeting the LH. This was followed by 7 days recovery and 10 days FR1 and 8 days FR3 training for sucrose. Mice were given a 7-day break from operant training to ensure the same timeline as Experiment 1. This was followed by 2 days of FR3 pre-stimulation training and 3 days of PR pre-stimulation training. Chronic PVN terminal optogenetic stimulation in the LH consisted of 5 days at 20 Hz, 10 ms pulse width, 15 mW, 30 s on 30 s off for 1 h/day. Stress-related behaviors were monitored during stimulation sessions for the first 10 min. Following stimulation, mice were exposed to 2 FR3 operant sessions and 3 PR sessions.

### Surgery

CRH-Cre or CRH-Cre::tdTomato mice were anesthetized with isoflurane (5% induction, 2% maintenance) before being placed into a stereotaxic frame (Stoelting Instruments). Mice received stereotaxic PVN-directed injections of either AAV5-DIO-ChR2-YFP or AAV5-DIO-YFP (Addgene; anteroposterior (AP): −0.7, mediolateral (ML): ± 0.25, dorsoventral (DV): −4.8) to selectively transduce CRH neurons (Fig. 1A, B). 0.2 µl was injected at a rate of 0.15 µl/min using a 2 µl Hamilton Neuros syringe (30 G) attached to a Stoelting Quintessential Stereotaxic Injector pump. Craniotomies were closed with BoneWax (Ethicon, NJ, USA) and the scalp incision secured with 3 M Vetbond and staples (AutoClip, Fine Science Tools, VIC, AUS) for Experiment 1 cohort 1. For the placement of fiber optic probes, two craniotomies were drilled and stainless-steel screws inserted (Avivamann Optical Group, WA, AUS). Probe and screws were secured to the skull with 3 M Vetbond and dental glue (Dentsply Sirona Pty Ltd, NSW, AUS). Mono fiber optic cannula placement (6 mm; 400 μm core; Slim Magnetic Receptor (SMR); 0.37 NA; Doric Lenses, QC, CAN) was either dorsal to the PVN and aimed toward the midline of the brain in Experiment 1 (AP: −0.7, ML: 0.0, DV: −4.3) or directly in the LH in Experiment 2 during the viral surgery (bilateral: AP: −1.2, ML: ±1.0, DV: −4.75) (Figs. 1A, 2A). After surgery, mice were given a subcutaneous injection of Ketoprofen (5 ml/kg) for analgesia.

### Sucrose self-administration

Mice were first trained to self-administer sucrose (10% w/v) in daily 30-min operant conditioning sessions under a fixed ratio 1 (FR1) schedule of reinforcement (Med Associates, VT, USA). A response on the active (right) lever resulted in the delivery of 10 µl of sucrose into a receptacle followed by a 4 s light cue located above the lever to indicate sucrose availability. Following 10 days of FR1 training, mice were moved to a FR3 schedule of reinforcement for the next 8 days where an inactive (left) lever was introduced which had no scheduled consequence (Fig. 1C). After FR3 training, mice had 7 days of rest from operant self-administration to allow for recovery from probe surgery from Experiment 1 cohort 1. This was followed by 2 additional FR3 operant sessions and 3 PR sessions. PR sessions occurred daily for 90 min using a reinforcement schedule where the number of lever presses required for sucrose reward delivery increased by 1 following each reward i.e. 1,2,3,4,…. etc. [26, 27]. PR breakpoint was defined as the highest response ratio completed. Both FR3 and PR testing were undertaken before and after repeated optogenetic stimulation (Figs. 1C, 2B). All behavioral testing took place between 8 am and 6 pm daily.

### In vivo optogenetics

Optogenetic experiments aimed to examine the behavioral activity and Fos-protein expression produced by the optogenetic activation of PVN^CRH^ neurons or PVN^CRH^ terminals in the LH [28]. One week prior to behavioral testing, animals were handled twice daily and habituated to their testing apparatus (Perspex arena; 42.2 cm L × 72 cm W × 60 cm H, with clean corn cob floor material). On the test day, animals were attached to fiber optic patch cords (400 μm core; FCM-SMC; 0.37 numerical aperture (NA); Doric Lenses) via their implanted mono fiber optic cannula. The opposing end of the patch cord was connected to a FC-FC fiber optic 1 × 1 rotary joint (Doric Lenses). A 473 nm DPSS laser (Laserglow Technologies, ON, CAN) was used to deliver light into the rotary joint via a patch cord with FC/FC connections on each end. Laser parameters were controlled using a Master-8 pulse stimulator (A.M.P.I, JRS, IL). Power output from the end of FCM-SMC patch cord (15 mW) was determined using a photodiode power sensor connected to a console (Thorlabs, NJ, USA). During each session, mice were given 30 min following patch cable attachment before the laser was turned on for 60 min.

### Acute behavioral analysis

For all animals, behavior was recorded for 12 min commencing 2 min before blue light stimulation (2 min baseline + 10-min laser stimulation analysis). Behavioral data was analyzed using a video annotator program (developed by Dr Toni-Lee Sterley – Hotchkiss Brain Institute, CAN) and used to quantify the duration of six distinct behaviors (digging, grooming, rearing, walking, jumping, inactive) [19]. Behavioral video analysis was undertaken by two individuals blind to treatment condition. The output from this program generated a record of all behaviors displayed over the 10-min period in Microsoft Excel.

### Immunohistochemistry and microscopy

Following the completion of the experiments, mice were euthanised with sodium pentobarbitone (0.2 ml intraperitoneal, I.P, Virbac, AUS) 2 h after the commencement of their final 1-h optogenetic stimulation session and perfused with 20 ml of 0.1 M Phosphate Buffer Solution (PBS) and 50 ml of 4% paraformaldehyde (PFA). Brains were extracted and postfixed in 4% PFA at 4 °C for 2 h, then transferred to 30% sucrose in 0.1 M PBS for 48 h at 4 °C. Brains were frozen on dry ice and stored at −80 °C until sectioning. Coronal sections 40 μm thick were cut on a cryostat (Leica Biosystems CM1900) in a 1 in 4 series and stored in a 0.1 M PBS solution containing 0.1% sodium azide at 4 °C. For the detection Fos-protein in Experiment 1, tissue sections were incubated in a solution of PBS containing 1% Triton X-100, 2% normal horse serum and primary antibody (1:8000, Phospho-c-Fos, rabbit monoclonal; Cell Signaling Technology, MA, USA, CAT# 5348) for 48 h at 4 °C. Sections were then washed 3 times for 10 min in PBS before incubation with the secondary antibody (1:1000, donkey anti-rabbit IgG, Jackson ImmunoResearch, PA, USA; Product code #711-005-152) for 2 h at room temperature. After washing to remove unbound secondary antibody, sections were incubated for 1 h using a Vectastain ABC kit (Vector Laboratories, CA, USA; SKU: PK-6100) followed by incubation with diaminobenzodine in 2% filtered nickel sulfate for 15 min. Visualization of Fos-protein was achieved by the addition of glucose oxidase. This tissue was then co-labeled for orexin-A. To co-label without cross-reactivity from rabbit polyclonal antibodies, an Avidin/Biotin Blocking kit was used (Vector Laboratories, CA, USA) before incubating tissue in orexin primary antibody (1:10000, rabbit monoclonal, Phoenix Pharmaceuticals, CA, USA; CAT # H-003-30) for 48 h at 4 °C. The procedures and reagents for visualization of cytoplasmic orexin immunoreactivity, ABC kit and visualization steps are the same as for Fos-protein with the exception that 2% filtered nickel sulfate was replaced with 0.1% acetate buffer. Following completion of immunohistochemical procedures, brain sections were mounted on chrome alum-treated slides, dehydrated in a series of ethanol and xylene solutions and coverslipped.

Photomicrographs of brain sections were made using Olympus CellSens Software (version 1.3) on an Olympus BX51 microscope at 10x objective. Bilateral cell counting was undertaken using iVision (Biovision) computer program for PVN slices (Bregma -0.6 mm to -0.8 mm). Image J was used to quantify Fos-positive and orexin-positive cells in LH sections (Bregma −1.22 to −1.7). Due to limited tissue availability, Fos-positive cells were also quantified in the globus pallidus externus (GPe; Bregma –0.7 to –0.82; YFP *n* = 12, ChR2 *n* = 13) and ventral tegmental area (Bregma –2.92 to –3.08; YFP *n* = 4, ChR2 *n* = 4) using ImageJ from a subset of animals. Coronal brain sections were chosen according to Paxinos and Franklin’s Mouse Brain in Stereotaxic Coordinates 6^th^ edition [29] and quantification was undertaken by one observer, blind to treatment.

PVN and LH sections from a second series of brain tissue were mounted onto microscope slides and coverslipped using a 0.1 M PBS and glycerol solution. Probe placement and viral expression were then visualized using an Olympus BX51 microscope. Please refer to Fig. S1 for fiber optic probe placements.

### Statistical analysis

Statistical analyses were conducted using JASP V16.2. Sucrose lever pressing was analyzed using an analysis of variance (ANOVA) with the between-subjects factor of Virus (YFP, ChR2) and the within-subjects factor of Day for initial FR1 and FR3 operant training, and two within subject factors, day and stimulation period (pre vs post) for FR3 and PR operant testing, as well as 5 min time bins for PR responding on Day 3. A two-tailed *t* test was used to assess the effect of optogenetic PVN^CRH^ neuron on single-measure data including recruitment on Fos-protein activity and day 3 PR. Pearson’s correlations examined the relationship between Fos activity in the LH and PVN of ChR2 animals. Stress-related behaviors were analyzed using ANOVA with the between-subjects factor of Virus (YFP, ChR2) and the within-subjects factor of Day. Tukey post-hoc comparisons were used to differentiate any significant interactions. The assumption of normality for ANOVA was analyzed using the Shapiro-Wilk test and the assumption of homogeneity of variances was assessed with Levene’s test of equality of variances. In some instances, these assumptions were violated however ANOVA is typically robust against these violations [30]. Tests of sphericity were assessed for within-subjects ANOVA comparisons, if sphericity was violated, Greenhouse-Geisser corrections were reported. Graphs show mean ± standard error of the mean (SEM).

## Results

### Effect of optogenetic recruitment of PVN^CRH^ neurons on motivation for sucrose

CRH-Cre transgenic mice were injected with a Cre-dependent excitatory opsin (ChR2) into the PVN (Fig. 1A, B). Additionally, a fiber optic probe was implanted immediately dorsal to the PVN to allow optogenetic activation of CRH neurons in the PVN (Fig. 1C). Mice were initially trained to lever press for sucrose by increasing fixed ratio schedules (Fig. 1C). Analysis of active lever presses during operant training revealed no significant interaction of Virus and Day during the FR1 schedule (*F*_9,234_ = 0.305, *p* = 0.973) or FR3 schedule of reinforcement (Fig. 1D; *F*_7,182_ = 0.731, *p* = 0.646). Following operant training, mice were assessed for their motivation for sucrose under both low effort (FR3) and high effort (PR) schedules of reinforcement at baseline and following 5 days of photostimulation of PVN^CRH^ neurons (Fig. 1C; “Stim” sessions). Under FR3 conditions, there was no effect of repeated PVN^CRH^ photostimulation on the number of active lever presses for sucrose (Fig. 1E; *F*_1,26_ = 0.315, *p* = 0.865). There was no effect of Virus on inactive lever presses during FR3 training days or PR sessions (Fig. S2). However, under higher effort PR conditions, there was a significant effect of repeated PVN^CRH^ photostimulation on the number of active lever presses for sucrose (*F*_2,52_ = 14.06, *p* < 0.0001). There was also an effect of repeated PVN^CRH^ stimulation on breakpoint during PR sessions (Fig. 1E; *F*_2,52_ = 14.20, *p* < 0.0001). Tukey post-hoc comparisons revealed significant differences in breakpoint between YFP control animals and ChR2 animals on Day 3 post-stimulation (Fig. 1F; *p* = 0.025). Closer inspection of 5 min time bin active lever press data on Day 3 post-stimulation revealed a significant interaction of Virus and Time bin on active lever presses (Fig. 1F; *F*_17,442_ = 5.105, *p* < 0.001). Interestingly, when the protocol was extended to 7 days of PR, in a second cohort of mice, the effect of repeated PVN^CRH^ stimulation on breakpoint persisted, but began to return to control levels by Day 7 (Fig. 1G; *F*_9,135_ = 3.379, *p* < 0.001).

### Effect of optogenetic recruitment of the PVN^CRH^ neurons on Fos-protein activity

On the day after the final PR session, mice underwent a final photostimulation session and were perfused 2 h later. Immunohistochemical analysis revealed an increased number Fos-positive cells in the PVN (*F*_1,26_ = 6.011, *p* = 0.021) and LH (*F*_1,26_ = 5.778, *p* = 0.024) in ChR2 mice compared to YFP controls (Fig. 1H; 1I). There was a trend for an effect of Virus on the percentage of Fos-positive orexin cells in the LH (Fig. 1J; *F*_1,26_ = 2.942, *p* = 0.098). An analysis of Fos-positive neurons in extra-hypothalamic areas revealed an increased number of Fos-positive cells in the GPe of ChR2 mice compared to YFP mice (Fig. S3A; *F*_1,23_ = 6.065, *p* = 0.022). There was no significant difference in the number of Fos-positive cells in the VTA of ChR2 mice compared to controls (Fig. S3B; *F*_1,6_ = 0.214, *p* = 0.660).

### Effect of optogenetic recruitment of the PVN^CRH^ neurons on stress-related behaviors

**Fig. 3:**
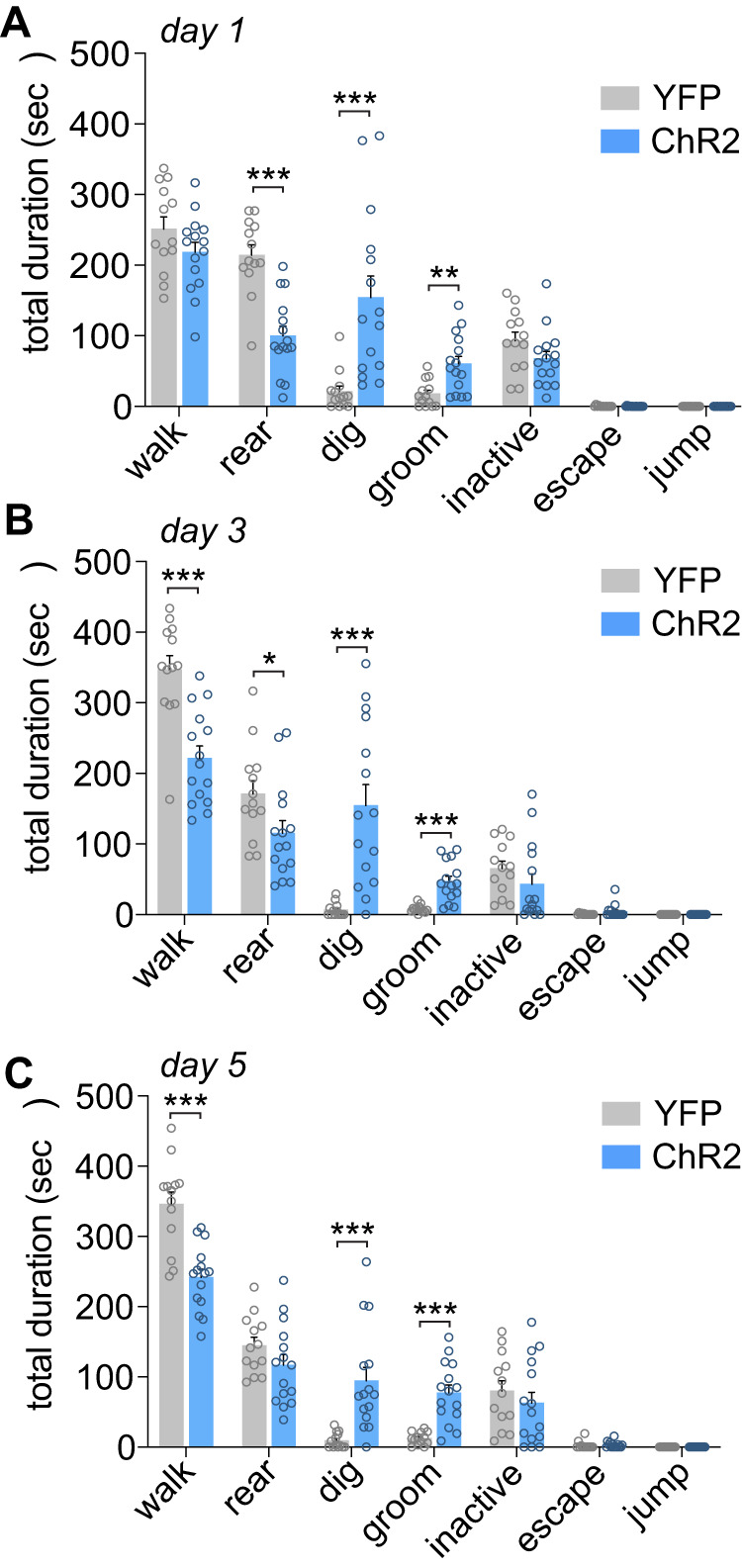
Optogenetic stimulation of PVN^CRH^ neurons increases stress-related behaviors. **A** During the first PVN^CRH^ photostimulation session, a shift in stress-related behaviors was observed in ChR2-mice compared to YFP controls including reduced rearing behavior, increased digging behavior and increased grooming. **p < 0.01, ***p < 0.001 post-hoc analyses. **B** During the third photostimulation session, ChR2 mice showed increased stress-related behaviors compared to YFP controls including reduced rearing behavior, increased digging behavior, increased grooming and reduced walking. *p < 0.05, ***p < 0.001 post-hoc analyses. **C** During the fifth PVN^CRH^ photostimulation session, ChR2 mice showed increased stress-related behaviors compared to YFP controls including increased digging behavior, reduced walking, and increased grooming. ***p < 0.001 post-hoc analyses. PVN paraventricular nucleus of the hypothalamus.

We determined the effect of PVN^CRH^ photostimulation on stress-like behaviors across the five days during the first 10 min of 473 nm blue light exposure. Behavioral analysis during the photostimulation sessions demonstrated a significant effect of Virus and Behavior on the time spent engaging in each stress-related behavior (Fig. 3A–C; *F*_6,189_ = 18.260, *p* < 0.0001). Tukey post-hoc comparisons showed that ChR2 mice exhibited significantly increased digging and grooming behaviors than YFP controls across the five sessions (*p*’s < 0.05). Additionally, ChR2 mice exhibited reduced rearing and exploratory behaviors (walking) compared to YFP controls (*p*’s < 0.05).

### Effect of optogenetic recruitment of the PVN^CRH^ to LH pathway on motivation for sucrose

Previous work has shown that the LH is a target of PVN^CRH^ terminals, and that activation of this pathway can elicit acute stress-related behaviors [19]. Further, in experiment 1 optogenetic photostimulation of PVN^CRH^ cell bodies resulted in increased LH Fos. Thus, we next asked whether repeated stimulation of this circuit would suppress the motivation for sucrose in a similar manner to cell body stimulation. CRH-Cre transgenic mice were injected with DIO-ChR2 into the PVN, and fiber optic probes were implanted bilaterally immediately dorsal to the LH (Fig. 2A, B). Mice completed the same sucrose training and photostimulation sessions as conducted in Experiment 1 (Fig. 2B), but instead, targeting PVN^CRH^ terminals in the LH. Analysis of active lever presses during operant training showed no significant effect of Virus and Day during the FR1 schedule (*F*_9,90_ = 0.713, *p* = 0.696) or FR3 schedule of reinforcement (Fig. 2C; *F*_7,70_ = 0.183, *p* = 0.988). Following operant training, mice were again assessed for their motivation for sucrose under both low effort (FR3) and high effort (PR) schedules of reinforcement at baseline and following 5 days of photostimulation of PVN^CRH^ terminals in the LH (Fig. 2B; “Stim” sessions). Under low effort, FR3 conditions, there was no effect of repeated PVN^CRH^ terminal photostimulation in the LH on the number of active lever presses for sucrose (Fig. 2D; *F*_1,10_ = 0.071, *p* = 0.795). There was also no significant effect of repeated PVN^CRH^ terminal stimulation in the LH on the number of active lever presses for sucrose under higher effort PR conditions (*F*_5,50_ = 1.569, *p* = 0.186). Following PVN^CRH^ terminal stimulation in the LH, there was a trend for altered breakpoint during PR sessions (Fig. 2D; *F*_2,20_ = 3.475, *p* = 0.051). Separate analysis of post-stimulation breakpoint revealed a significant Day × Virus interaction (*F*_2,20_ = 4.718, *p* = 0.021), suggesting a similar pattern of effects to cell body stimulation in Fig. 1F. The largest effect of PVN^CRH^ photostimulation observed in Experiment 1 occurred on Day 3 of PR. Thus, we also decided to closely examine this time point following optogenetic recruitment of the PVN^CRH^ to LH pathway. There was no significant effect of PVN^CRH^ terminal stimulation in the LH on PR breakpoint when comparing pre-stimulation versus post-stimulation on Day 3 of PR testing (Fig. 2E; *F*_1,10_ = 2.781, *p* = 0.126). There was a trend towards a significant reduction in the number of active lever presses for sucrose in ChR2 mice compared to YFP controls on PR Day 3 post-stimulation (Fig. 2F; *F*_1,10_ = 3.517, *p* = 0.090). Time bin active lever press data on Day 3 post-stimulation revealed a significant effect of Virus and Time bin on active lever presses (Fig. 2G; *F*_17,170_ = 2.782, *p* < 0.001).

### Effect of optogenetic recruitment of the PVN^CRH^ to LH pathway on stress-related behaviors

**Fig. 4:**
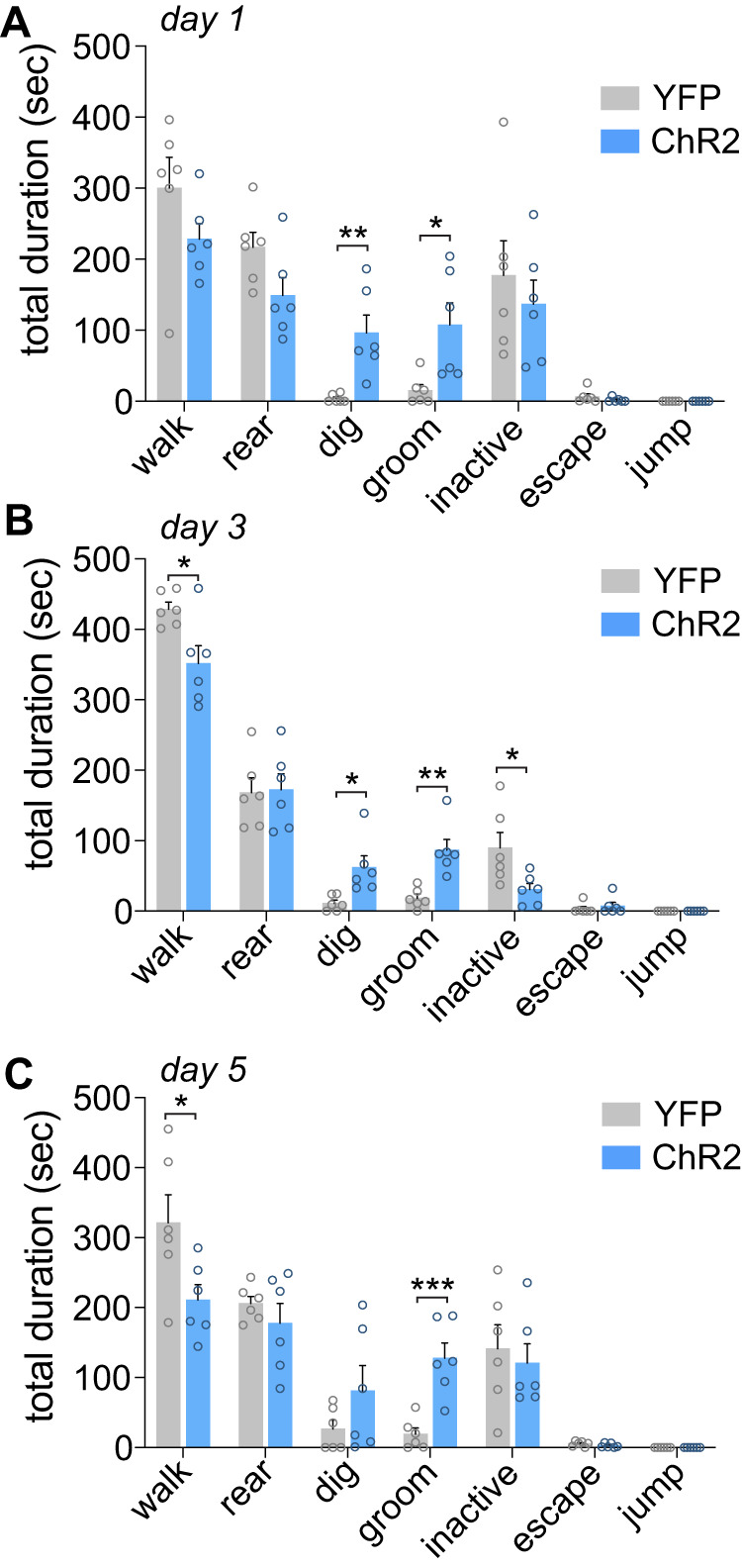
Optogenetic stimulation of the PVN^CRH^ to LH pathway increases stress-related behaviors. **A** During the first photostimulation session, ChR2 mice showed increased stress-related behaviors compared to YFP controls including increased digging behavior and increased grooming. *p < 0.05; **p < 0.01 post-hoc analyses. **B** During the third PVN^CRH^ to LH pathway photostimulation session, ChR2 mice showed increased stress-related behaviors compared to YFP controls with increased grooming behavior, increased digging, reduced walking and reduced periods of inactivity. *p < 0.05; **p < 0.01 post-hoc analyses. **C** During the fifth photostimulation session, ChR2 mice showed increased grooming behavior and reduced walking. *p < 0.05; ***p < 0.001 post-hoc analyses. PVN paraventricular nucleus of the hypothalamus, LH lateral hypothalamus.

Similar to PVN^CRH^ stimulation experiments, optogenetic recruitment of the PVN^CRH^ to LH pathway significantly increased stress-related behaviors compared to YFP controls. There was a significant effect of Virus and Behavior on the time spent engaging in each stress-related behavior (Fig. 4A–C**;** *F*_6,70_ = 8.24, *p* < 0.0001). Tukey post-hoc comparisons showed that ChR2 mice had significantly increased digging during the stimulation on day 1 compared to YFP controls (*p* < 0.05). Additionally, ChR2 mice exhibited increased grooming across the three sessions analyzed (p’s < 0.05). There was a significant correlation between PVN Fos activity and LH Fos activity in ChR2 mice, (Fig. S4; *r* = 0.817, *p* < 0.0002).

## Discussion

Stress-induced activation of the HPA axis is strongly associated with mood disorders such as depression, however, a direct link between the activation of PVN^CRH^ neurons that control the HPA axis and impairments in motivational drive has been lacking. Here we show that repeated photoactivation of PVN^CRH^ neurons increased acute stress-related behaviors including grooming and digging, and is sufficient to reduce the motivation to lever press for sucrose, exclusively in a high-effort progressive ratio schedule of reinforcement. Interestingly, this effect persisted for up to 7 days following PVN^CRH^ stimulation. Direct stimulation of PVN^CRH^ neurons increased Fos-protein activity in the PVN and LH, with a trend towards an increase in LH orexin-expressing neurons. Given that recent literature has implicated the PVN^CRH^ to LH pathway in stress-related behaviors and early life stress promotes LH-driven impairments in motivational drive [19, 20], we examined the direct effect of PVN^CRH^ to LH stimulation on the effort to lever press for sucrose. Repeated activation of this pathway produced a similar pattern of changes in PR responding to PVN^CRH^ neuronal cell body stimulation, although not as robustly as activating PVN^CRH^ neuron cell bodies directly. Acutely, photoactivation of PVN^CRH^ terminals in the LH also produced characteristic stress-associated behavioral responses, including increased digging and grooming. Together, these data suggest that PVN^CRH^ neuron activity can precipitate depressive-like behaviors that may extend beyond increased HPA axis activation, as demonstrated by our finding that repeated photostimulation of PVN^CRH^ neurons directly impacts motivational drive.

### PVN^CRH^ activation increased acute stress-related behaviors, reduced motivated responding for sucrose, and altered Fos-protein immunoreactivity

In the current study, we found that repeated activation of PVN^CRH^ neurons selectively suppressed responding for sucrose under a high-effort progressive ratio (PR) schedule of reinforcement, but not under low-effort fixed ratio schedule (FR3). This effect of PVN^CRH^ stimulation on motivational drive persisted for up to 7 days post-stimulation, although the effect weakened over time. This finding demonstrates that repeated activation of PVN^CRH^ neurons has a lasting impact on the incentive motivation for sucrose, a phenotype typically observed in people with depression. Whilst we cannot discount the impact of repeated PVN^CRH^ activation on other cognitive domains (particularly those associated with depressed states) [31], an effect of repeated stimulation on FR3 responding was not evident, suggesting relatively selective effects on high effort motivation. Additionally, prior to PVN^CRH^ stimulation, both YFP controls and ChR2 mice showed no differences in FR1 or FR3 operant training, arguing against any potential unexpected behavioral effects of the virus. Interestingly, the impact of stimulation on sucrose responding only began to manifest from day 2 of PR testing. We have observed similar behavioral effects in our previous work assessing the impact of early life stress on the motivation for sucrose [20]. In this study, PR breakpoints were similar on day 1 of testing in control and early life stressed groups, however stressed rats showed a significant decrease in motivation from day 2 onwards. This delayed effect may reflect a loss of capacity to maintain sustained motivation over repeated sessions. This phenotype is consistent with other preclinical work demonstrating that social defeat stress reduces PR responding for saccharin and sucrose consumption up to 5 weeks following stress [32, 33].

In the current study, the motivational performance of the ChR2 group recovered after 7 days of PR testing, suggesting the possibility of compensatory re-engagement in the task. The effects also suggest that 5 days of PVN^CRH^ photostimulation are not sufficiently intense to induce “permanent’ changes in circuit function, however a longer-term exposure may do so. Indeed, for many chronic stress paradigms, several weeks of stress are required to see long-lasting changes in behavior and physiology [34]. Analyzing protracted time points within the experiment and assessing individual susceptibility to repeated PVN^CRH^ photostimulation may also be relevant for more direct comparisons to depression-related phenotypes. Regardless, our preclinical findings and previous research align with the human depression literature where chronic stressors have been associated with persistent depressive symptoms, frequent relapse, and poorer prognosis [35, 36]. Importantly, our findings suggest a direct role for PVN^CRH^ neurons in these depression-related motivational impairments.

Consistent with recent work [19], we found that optogenetic activation of PVN^CRH^ neurons produced a highly reproducible pattern of stress-related behaviors. We extend this work by demonstrating that these PVN^CRH^ stimulation-evoked behaviors are relatively stable across five days of stimulation for certain behaviors such as grooming and digging. This pattern was characterized by increased grooming and digging, along with reduced walking and rearing behavior, although a loss of reductions in rearing was observed on Day 5. Grooming is a complex innate behavior and in the context of stress is thought to be important for de-arousal following stress exposure [37]. The increase in digging behavior we observed during activation of PVN^CRH^ neurons has not been previously reported following a similar experimental design [19]. A potential explanation for digging in our experimental preparation is the inclusion of bedding in the test cage, which may have allowed for the expression of this behavior. Like grooming, digging is an innate rodent behavior that has potential for pathological repetition and is associated with stressful and anxiogenic situations [38]. Digging has conceptual similarity to the anxiety-associated marble burying test, which has been argued to be more appropriately considered an indicative measure of repetitive digging [39, 40]. However, it is important to note that behavioral effects following repeated photostimulation may also be reflective of dysregulated motor behavior. Changes in walking or rearing may be relevant to stress-induced exploratory behavior, or hypervigilance for threat assessment. Interestingly, the current behavioral data and that of Füzesi and colleagues [19] suggests that optogenetic stimulation of PVN^CRH^ neurons does not fully recapitulate typical behavioral patterns following acute stress, such as increases in walking and rearing [19]. Instead, PVN^CRH^ photostimulation may drive complex behavioral selection in which repetitive, internally focused stress behaviors predominate. Importantly, behaviors elicited by optogenetic stimulation occurred rapidly, within seconds, therefore these responses are likely driven by synaptic rather than neuroendocrine mechanisms [41]. This is supported by our similar acute behavioral observations following selective optogenetic stimulation of PVN^CRH^ terminals in the LH. These findings indicate that PVN^CRH^ neurons mediate fast, stress-relevant behaviors, likely independent of hormonal feedback [19].

Following repeated PVN^CRH^ photostimulation, we observed a significant increase in the number of Fos-positive cells in the PVN and LH. These findings are consistent with work from Füzesi and colleagues [19], who showed that acute activation of PVN to LH terminals can produce a range of stress-relevant behaviors through direct, excitatory projections to the LH. However, the identity of these LH Fos-positive neurons remains to be determined. Prior evidence from our lab has shown a role of LH orexin in early life stress-induced motivational deficits [20], and in the current study repeated PVN^CRH^ stimulation was associated with a trend towards a significant increase in the percentage of Fos-positive orexin cells. This is interesting as other work using monosynaptic retrograde rabies tracing has shown that CRH directly innervates LH orexin neurons [42]. While this effect did not reach statistical significance, there are known limitations with Fos-protein mapping including sensitivity and temporal resolution. Additionally, orexin neurons are spontaneously active and sensitive to many different environmental and behavioral factors, including changes in light, social interaction and energy status [43]. Future studies using electrophysiological recordings or Ca^2+^ imaging might be required to detect temporally specific changes in orexin cell activity [19, 20]. Finally, we cannot rule out the possibility that other LH neuronal populations may be playing a role including melanin-concentrating hormone neurons and GABAergic neurons [44–46].

### PVN^CRH^ to LH pathway activation modestly reduced motivated responding for sucrose and increased stress-related behaviors

A similar, although weaker, effect on high-effort PR responding was found following repeated photostimulation of PVN^CRH^ terminals in the LH. Photostimulation of PVN^CRH^ terminals in the LH also produced a similar pattern of acute stress-related behaviors, including digging and grooming, as PVN^CRH^ cell body stimulation, consistent with previous literature [19]. There is growing interest in the projections of PVN^CRH^ neurons beyond the median eminence, with recent demonstrations of important functions outside HPA axis and autonomic activity. Indeed, PVN^CRH^ neurons are known to project to a number of other downstream sites with well described roles in reward and stress-coping behaviors, such as the ventral tegmental area (VTA), locus coeruleus, periaqueductal gray and globus pallidus [47–52]. Thus, it may be that activation of additional terminal projections of the CRH neurons is required to produce the full PVN^CRH^ neuronal cell body stimulation effect on motivation [53]. In support, acute stress results in CRH release in the VTA, where it has been shown to modulate reward-related behavior [54]. Thus, future research on motivational drive following activation of other reward-related projection pathways of PVN^CRH^ neurons is required.

### Methodological considerations

Our most interesting finding is that direct PVN^CRH^ stimulation produces persistent reductions in motivational drive and an immediate onset of stress-related grooming and digging. This appears to be independent of HPA-axis activity and extends the previous work of Füzesi and colleagues [19]. One limitation of the current study is that we did not assess the involvement of circulating glucocorticoids on motivational drive. Whilst the current experiments do not have a direct read-out of neuroendocrine output, we know CRH neuron activation leads to glucocorticoid secretion, but given the reciprocal connections with the LH we cannot rule out the effects of LH terminal stimulation on circulating glucocorticoids [55, 56]. Importantly, the key outcome from this work is that activation of CRH neurons in the PVN is sufficient on its own to promote motivational disturbances, suggesting that future studies examining the links between stress and behavior need to consider both the synaptic and endocrine projections of these cells.

Another important consideration is the selectivity of AAV-ChR2 to CRH neurons within the PVN. The CRF-Cre mouse line has been validated by previous work, using an overlap of endogenous CRH-tagged td::tomato and PVN-specific Fos expression following an acute stressor [19, 25]. However, there are non-CRH expressing neurons located within the PVN, i.e. the magnocellular neurons that synthesize and release oxytocin and vasopressin [57]. The current methodologies cannot completely rule out non-selective and/or indirect ChR2 activation within these populations; however, this is a potential limitation of many optogenetic studies. Future experiments using electrophysiological patch-clamp recordings within PVN slices to study local connectivity will be important to identify possible contributions of other cell types involved. An additional consideration is the potential for neuronal damage due to the repeated nature of the optogenetic stimulation. The strength of the photostimulation in the current design (15 mW) is based on published optogenetic work [19], and the potential for tissue overheating is mitigated by the use of a pulse train and 30 s cycles of pulses followed by 30 s of no laser stimulation, as opposed to employing a continuous light signal [58]. No obvious tissue damage was observed during cFos processing of brain slices, however additional immunohistochemical markers sensitive to cell death should be considered for future experiments.

## Conclusions

Here we show ChR2-mediated stimulation of PVN^CRH^ neurons produced a long-lasting reduction in the motivation for a natural reward, distinct from a general behavioral deficit in sucrose operant responding as demonstrated by lack of effects on low effort behavior. These results are consistent with literature suggesting that repeated activation of stress responses mediated by the PVN can lead to depressive-like behaviors in rodents and humans [59, 60]. Overall, these data have important implications for the identification of novel hypothalamic circuits that govern stress-induced changes in mood and motivation. These current advances may aid in developing more effective treatments for debilitating symptoms of neuropsychiatric diseases such as depression, through selective targeting of these PVN projection pathways.

### Supplementary information

Supplementary material

## Data Availability

All data are available upon request to the corresponding authors
