# Supplementary material for: Optogenetic recruitment of hypothalamic corticotrophin-releasing-hormone (CRH) neurons reduces motivational drive
Source: Transl Psychiatry. 2024 Jan 8;14:8. doi: 10.1038/s41398-023-02710-0 (PMC10774335; doi:10.1038/s41398-023-02710-0)
Supplement: Supplementary file 1 — Supplementary material [file 41398_2023_2710_MOESM1_ESM.pdf]

Supplementary Figures

Optogenetic recruitment of hypothalamic corticotrophin-releasing-hormone (CRH) neurons reduces motivational drive

Caitlin S Mitchell[1,2†], Erin J Campbell[1,2†], Simon D Fisher[1,2], Laura M Stanton[1,2], Nicholas J Burton[1,2], Amy J Pearl[1,2], Gavan P McNally[3], Jaideep S Bains[4,5], Tamás Füzesi[4,5], Brett A Graham[1,2], Elizabeth E Manning[1,2*], Christopher V Dayas[1,2*]

[1]School of Biomedical Sciences and Pharmacy, Faculty of Health and Medicine, University of Newcastle, Callaghan, NSW, 2308, Australia

[2]Brain Neuromodulation Research Program, Hunter Medical Research Institute, New Lambton Heights, NSW, 2305, Australia

[3]School of Psychology, University of New South Wales, UNSW, Sydney, NSW, 2052, Australia

[4]Hotchkiss Brain Institute, Cumming School of Medicine, University of Calgary, Calgary, Alberta, Canada

[5]Department of Physiology and Pharmacology, Cumming School of Medicine, University of Calgary, Calgary, Alberta, Canada

[†] Contributed equally
*Co-senior authors

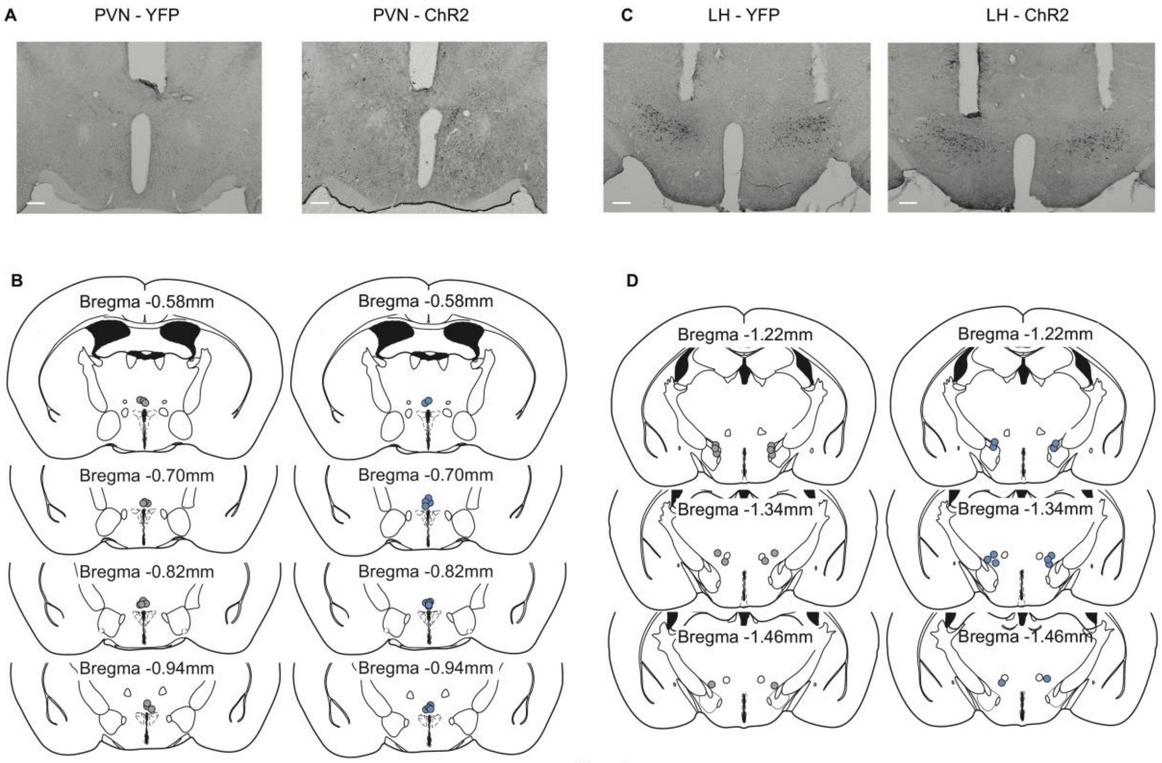

Supp.1

Figure S1. Fibre optic probe placement for PVN^{CRH} stimulation experiments and PVN^{CRH} to LH experiments.

A, Photomicrographs representing fibre optic probe placement for PVN stimulation. **B**, Fibre optic probe placement above the PVN for YFP control mice and ChR2 mice for repeated PVN^{CRH} stimulation experiments. **C**, Photomicrographs representing dual fibre optic probe placement for LH stimulation. **D**, Fibre optic probe placement relative to Bregma above the LH for YFP control mice and ChR2 mice for repeated PVN^{CRH} to LH stimulation experiments. Mouse brain coordinates were based on Paxinos & Franklin, 2001. PVN, paraventricular nucleus of the hypothalamus; LH, lateral hypothalamus. Scale bar = 1000µm.

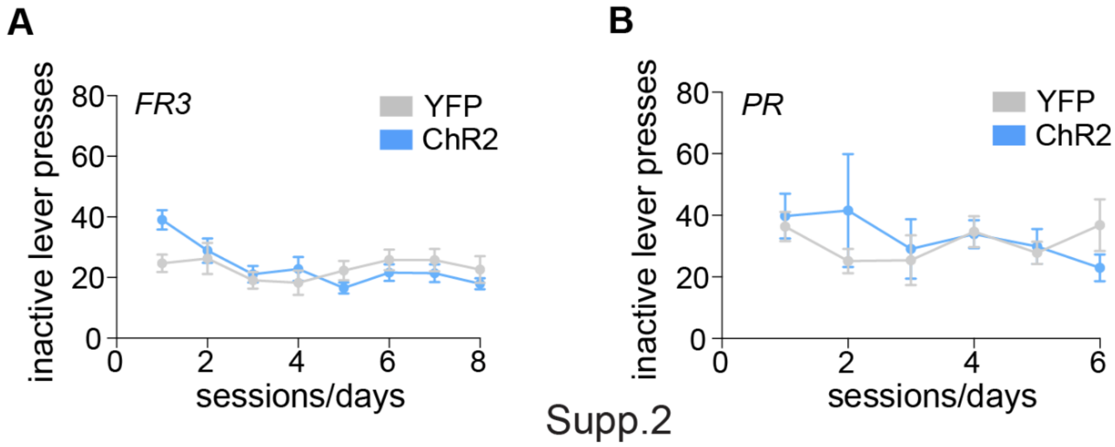

Supp.2

Figure S2. Optogenetic stimulation of PVN^{CRH} neurons does not impact inactive lever pressing during operant training for sucrose.

A, There was no difference between mice injected with YFP control virus versus ChR2 virus in the number of inactive lever presses across FR3 training days. **B**, There were no differences in the number of inactive lever presses during PR training between YFP control mice and ChR2 mice (sessions 1-3). There was also no effect of repeated PVN^{CRH} photostimulation on the number of inactive lever presses during PR sessions between YFP control mice and ChR2 mice (sessions 4-6). Progressive ratio (PR); fixed ratio (FR).

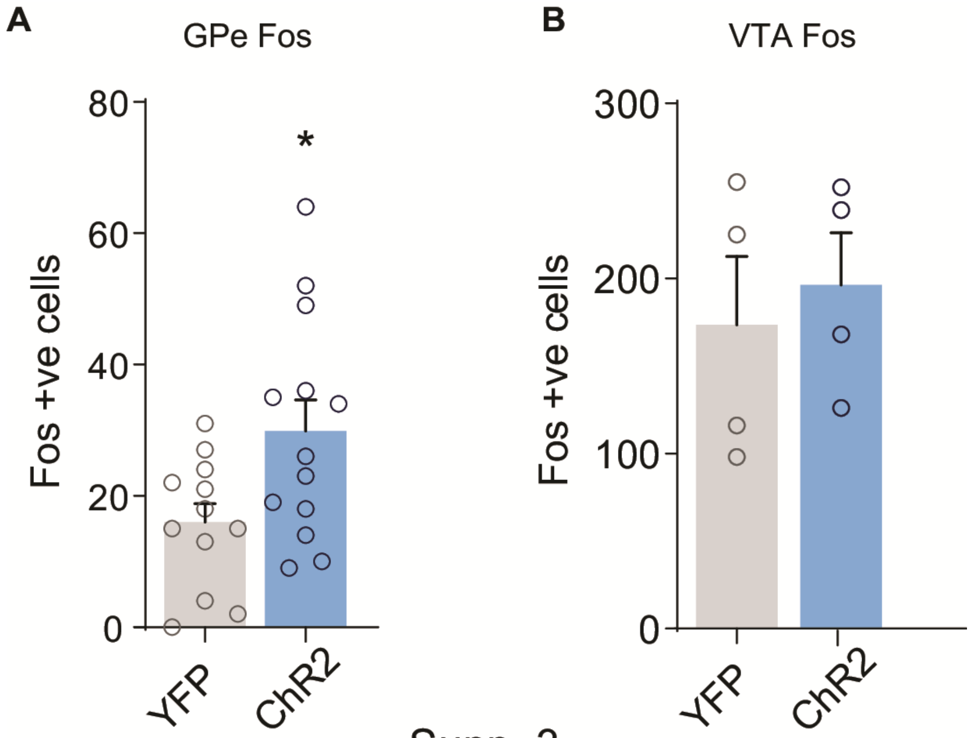

Supp. 3

Figure S3. Repeated optogenetic stimulation of PVN^{CRH} neurons increases Fos-protein expression in the GPe of ChR2 mice compared to YFP controls.
A, There was a significant increase in the number of Fos-positive cells in the GPe in ChR2 mice compared to YFP controls following PVN^{CRH} stimulation, *$p<0.05$. **B**, There was no significant difference in the number of Fos-positive cells in the VTA between treatment groups.

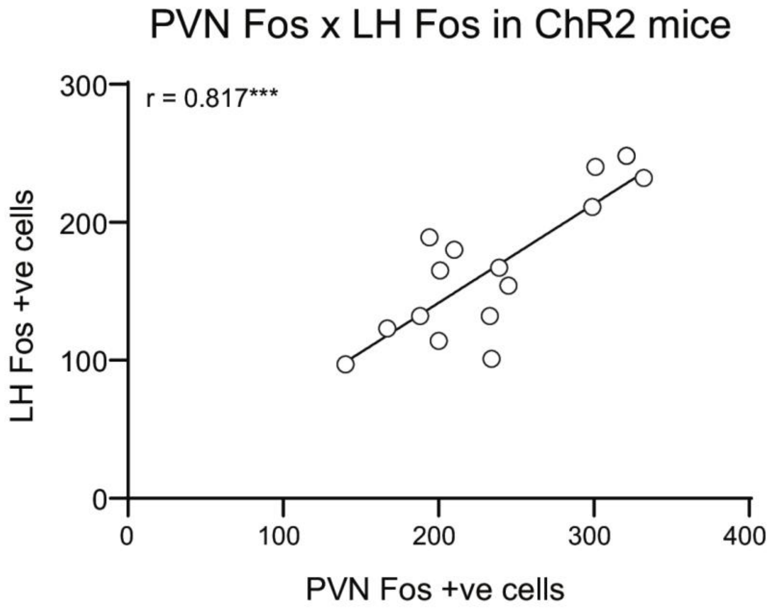

Supp. 4

Figure S4. Correlation between PVN Fos and LH Fos following repeated optogenetic stimulation of PVN^{CRH} neurons in ChR2 mice. Pearson's correlations showed a significant relationship between Fos activity in the LH and PVN in ChR2 animals, $p < 0.001$.
